# Recovery of brain biomarkers following peroxisome proliferator-activated receptor agonist neuroprotective treatment before ischemic stroke

**DOI:** 10.1186/1477-5956-12-24

**Published:** 2014-05-06

**Authors:** Patrick Gelé, Valérie Vingtdeux, Camille Potey, Hervé Drobecq, Antoine Ghestem, Patricia Melnyk, Luc Buée, Nicolas Sergeant, Régis Bordet

**Affiliations:** 1 Clinical Investigation center, IMPRT, University of Lille II, Cardiologic Hospital, Lille, France; 2 Inserm UMR 837, JPARC, Place de Verdun, Lille 59045, France; 3 PRES University Lille Nord de France, University of Lille II, Jean-Pierre Aubert Research Center, Institute of Predictive Medicine and Therapeutic Research, Lille IFR114, France; 4 UMR 8161 CNRS, Biomolecules and Micro-nanotechnologies laboratory - University of Lille 2 - University of Lille 1 – Pasteur Institute of Lille, Lille, France; 5 EA1046 - Department de Pharmacology - University of Lille 2, University Hospital Centre Place de Verdun, Lille, France

**Keywords:** Stroke, Statin, Fibrate, Neuroprotection, Proteomics

## Abstract

**Background:**

Lipid lowering agent such as agonists of peroxisome proliferator-activated receptors (PPAR) are suggested as neuroprotective agents and may protect from the sequelae of brain ischemic stroke. Although the demonstration is not clearly established in human, the underlying molecular mechanism may be of interest for future therapeutic purposes. To this end, we have used our well established rodent model of ischemia-reperfusion pre-treated or not with fenofibrate or atorvastatin and performed a differential proteomics analyses of the brain and analysed the protein markers which levels returned to “normal” following pre-treatments with PPARα agonists.

**Results:**

In order to identify potential therapeutic targets positively modulated by pre-treatment with the PPARα agonists, two-dimensional gel electrophoresis proteome profiles between control, ischemia-reperfusion and pre-treated or not, were compared. The polypeptide which expression was altered following ischemia – reperfusion but whose levels remain unchanged after pre-treatment were characterized by mass spectrometry and further investigated by Western-blotting and immunohistochemistry. A series of 28 polypeptides were characterized among which the protein disulfide isomerase reduction – a protein instrumental to the unfolded protein response system - was shown to be reduced following PPARα agonists treatment while it was strongly increased in ischemia-reperfusion.

**Conclusions:**

Pre-treatment with PPARα agonist or atorvastatin show potential neuroprotective effects by inhibiting the PDI overexpression in conjunction with the preservation of other neuronal markers, several of which are associated with the regulation of protein homeostasis, signal transduction and maintenance of synaptic plasticity. This proteomic study therefore suggests that neuroprotective effect of PPARα agonists supposes the preservation of the expression of several proteins essential for the maintenance of protein homeostasis not necessarily directly linked to PPARα known-regulated targets.

## Introduction

Ischemic stroke is the second leading cause of death and the first one of disability in adults of industrialized countries [1], [2]. In the acute phase of stroke, there is currently one major therapeutic strategy using the tissue plasminogen activator of thrombolysis [3], [4]. To date, clinical trials have not demonstrated any neuroprotective effect of a single drug at the acute phase of ischemic stroke. Thus, the interest has turned to alternative therapeutic strategies such as preventive neuroprotection based on the induction of cerebral protection to ischemia prior to its occurrence. For that purpose, two families of antilipidemic drugs, including fibrate and statin families likely exhibit a neuroprotective effect in an experimental model of ischemic stroke [5], [6]. The clinical demonstration of efficacy of fibrate in stroke is still under investigation [7] whereas statin therapy is more promising [1] and even recommended for elderly patients who recently had a stroke or transient ischemic attack [8].

The two families molecules have different signaling pathway and their use in stroke therapy is based on their neuroprotective way of action. The fibrate family contains several compounds that are all peroxisome proliferator-activated receptor alpha (PPARα) agonists. PPARα is one of the three subtypes of the nuclear receptor PPAR that is essential to regulation energy metabolism and vascular homeostasis [9], and is, as the β/δ and γ subtypes, expressed in brain tissue [10]. Experimental data strongly suggest that PPARs are interesting targets in ischemic stroke [11]. The activation of PPARα by fibrates has been demonstrated to induce neuroprotection, by reducing the brain infarct volume [12] and by the modulation of several pathophysiological pathways: inflammation, oxidative stress, the amyloid cascade [13]–[16]. The activation of PPARα by its ligands stimulates target gene transcription via the formation of a heterodimer complex of transcription factor with the retinoid X receptor (RXR) [17]. In the brain, PPARα activation leads to the expression of target genes such as Cu/Zn SOD or glutathione peroxidase [18], [19] resulting in an increased redox state. Moreover, PPARα activation represses NF-κB and the activator-protein-1 signaling pathways [20] thereby down regulating the inflammatory response and oxidative stress. Moreover, Brain activation of PPARα has been described as potentially neuroprotective in several models of brain pathologies such as ischemic stroke or closed head injury [21], [22] and this effect is abrogated by co-treatment by a PPARα antagonist [12].

Statins were firstly developed as molecular inhibitor of the hydroxyl methyl coenzyme A (HMG-CoA) reductase, a key enzyme of cholesterol synthesis. Statins reduce synthesis of endogenous cholesterol and isoprenoids, molecules that modulate several cell functions [23]. Thus, Chen and collaborators had shown that statin permit an improved functional recovery after stroke through a statin-induced amplification of angiogenesis, neurogenesis and synaptogenesis [24], [25]. The mechanisms underlying such effect are multiple and in relationship with the pleiotropic effect of statins. For instance, statins activates the PI3kinase/AKT and RAS/ERK pathways [26]. Phosphorylation of AKT and ERK transduces cell-surviving signal [27]. These pleiotropic effects are, at least partly, mediated by the activation of PPARα [16]–[28] and hence, the effect of statins could overlap that of PPARα agonists.

Due to the pleiotropic molecular cascades induced by those two pharmacological classes of hypolipidemic drugs associated to their potential usefulness as neuroprotective drugs in stroke, we investigated the modifications of the brain proteome induced the pre-treatment with those drugs before brain ischemia/reperfusion. This proteomics analysis was performed in our model of brain ischemia-reperfusion pre-treated or not with fenofibrate and atorvastatin. Using this approach we aimed to identify metabolic pathway stimulate by these drugs and associated to neuroprotection.

## Results and discussion

### 2D Brain proteome analysis

**Figure 1:**
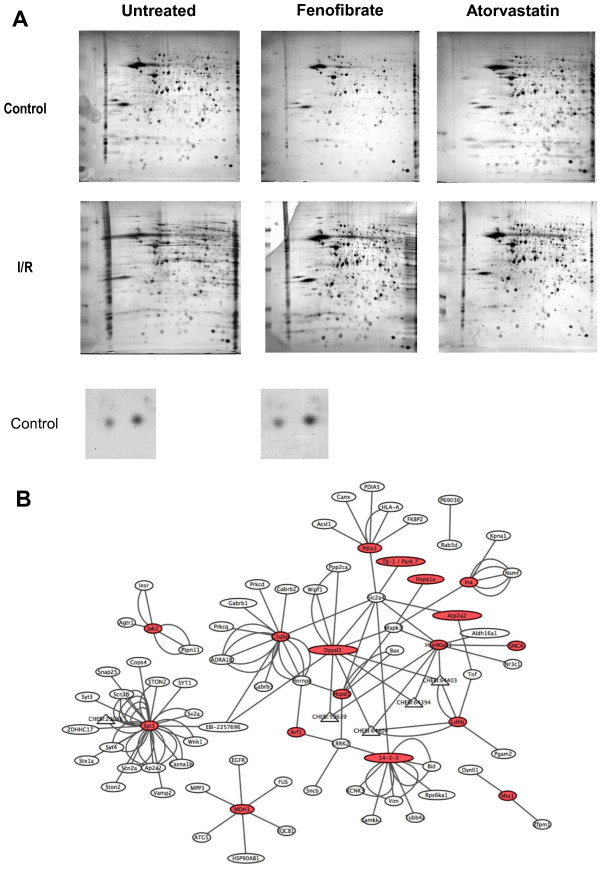
**Proteomics analysis of cortex from hypolipidemic drugs - treated rats under control or ischemia**-**reperfusion conditions. A**. The shown gels are representative of the observed results. Each condition was reproduced at least three times before spots excision and MALDI-TOF analysis. 6 animals per group were included. **B**. Representation of the interactome obtained with CytoScape Freeware. The markers identified are colored in red.

For this assay, animals were divided in six experimental groups: control group, fenofibrate treated group, atorvastatin treated group, ischemia-reperfusion injury group, fenofibrate treated and ischemia-reperfusion injury, atorvastatin treated and ischemia-reperfusion injury. In order to potentially identify the major differences in between groups and not the heterogeneity in between animals, the brain tissues of the animals of each group were pooled and processed for 2D analysis. The 2D gels were repeated a minimum of three times in order to obtain at least three gels that could be analyzed using Melanie III Software. Gels were successively stained with Coomassie blue and silver staining before being digitized. Examples of 2D profiles of rat brain proteomes are shown on Figure 1. The overall profile is reproducible in between experimental conditions showing that in the ischemia-reperfusion conditions, the brain proteome is not altered by the experimental procedure. Therefore, 2D gels were compared in between control and ischemia-reperfusion and in groups of animals treated or not with drugs. Gels were analyzed with Melanie III software and differences were processed for mass-spectrometry identification. Three kinds of differences were observed such as an increased or decreased expression or isoelectric point variations when compared to the control condition. Three series of 28 polypeptides were isolated and characterized following in-gel trypsin-digestion of the selected spots and mass spectrometry identification (Table 1). In order to identify the protein markers modulated following therapeutic treatments in cerebral ischemia-reperfusion injury, two sets of experimental conditions were tested. First, we analyzed the mismatches between the control group and the ischemia-reperfusion injury group. Second, in order to identify potential therapeutic targets for neuroprotection, we attempted to identify variations between ischemia-reperfusion conditions with or without the neuroprotective preconditioning treatments. Moreover, variations of the proteome profile should have been comparable to that observed in the control proteome pattern. The modifications observed are summarized in the last column of Table 1.

**Table 1:** **Proteins identified by MALDI**-**TOF analysis**

**Accession number**	**Protein**	**Function**	**pI/MW (kDa)**	**Peptides match/%**	**Variation**
P35213	14-3-3 isoform zeta, PKC inhibitor	Signal transduction	5.5/28	9/40	↑ I/R, ↑ Atorva
P35213	14-3-3 protein beta/alpha	Signal transduction	4.8/28	7/31	↑ Atorva, ↓ Feno
P45953	Acyl-CoA dehydrogenase, very long chain specific	Lipid Metabolism, Mito	9.0/70.8	4/16	↑ I/R
P84079	ADP rybosilation factor 1	Trafficking	6.3/20.7	4/16	↑ I/R
Q9Z0U5	Aldehyde oxidase precursor	Redox regulation	6.5/146.9	5/25	↑↑ I/R, ↑I/R Atorva, ↑, I/R Feno
P37840	Alpha-synuclein	Presynaptic/axon	4.7/14.5	4/11	↓ I/R, ↓ Atorva
P23565	Alpha-internexin	Intermediate filament	5.2/56.2	5/26	↓ I/R
Q63754	Beta-synuclein	Neuronal plasticity	4.5/14.3	5/17	↑ I/R
Q05175	Brain acid soluble membrane protein (NAP 22)	Growth cone/axon	4.5/21.8	4/15	↑ I/R
O88767	DJ-1	Pleiotropic	6.3/20	5/31	↓ I/R
P11598	Glucose related protein 58 kDa/PDIA3/Erp60	Thioredoxin/protein folding	5.9/57.6	4/17	↑ I/R, ↓ Atorva
O35077	Glycerol-phosphate-deshydrogenase	Metabolism	6.3/38	2/11	↑ I/R
O35796	GCQ1QBP Glycoprotein	Complement/Mito	4.8/30.7	4/21	↓ I/R
P63039	HSP 60	Protein folding	8.2/58.08	5/14	↑ I/R
Q07439	HSP 70.1/2	Protein folding	5.5/70.3	3/14	↓ I/R
P34058	HSP 90 beta	Protein folding	5.1/83.4	7/38	↑ Feno
Q62689	Janus Kinase 2	Tyrosine kinase/inflammation	7.1/130.5	7/30	↓ I/R, ↑ Atorva
P42123	Lactate dehydrogenase B chain	Metabolism	5.7/36.6	6/25	↓ I/R
P40925	Malate dehydrogenase	Metabolism	6.2/36.7	3/15	↑ I/R
Q62599	Metastasis associated protein 1	Unknown brain function	9.2/79.4	4/26	↓ I/R
P52590	Nucleoporine 107	Nuclear pore	5.3/107.2	4/26	↓ I/R
P35704	Peroxiredoxine 2	Redox regulation	5.3/21.8	6/42	↑ Feno
P31044	Phosphatidylethanolamine-binding protein	Serine protease inhibitor	5.5/20.8	6/17	↓ I/R
P35291	Rab16	Trafficking	5.0/22	4/25	↑ Atorva, ↑ Feno
P49803	Regulator of G protein signaling 7	Signal transduction	8.3/55.7	4/50	↑ I/R
P11507	Serca 2	Calcium Homeostasis	5.2/114.7	12/14	↓ I/R
P21707	Synaptotagmin I	Synapse	8.8/47.8	4/22	↑ I/R
P47942	TUC-4 / TOAD 64 (DYPL/CRMP)	Axonal guidance/development	6.0/62.2	15/71	↑ I/R

A major part of the proteins identified after 2D analysis were submitted to variation 24 hours after cerebral ischemia-reperfusion injury (24 among the 28 polypeptides characterized) when compared to the control group. The most representative variation observed is an increase of expression in the ischemia-reperfusion group. Among the proteins increased in expression, several are involved in the energetic metabolism of the cell such as aldehyde oxidase precursor, or malate dehydrogenase precursor. A second group was constituted with proteins involved in intracellular signaling pathways: i.e. 14-3-3 ζ as PKC inhibitor, or the regulator of G protein signaling 7. Other proteins are localized to the synapse and are important structural proteins, such as α-synuclein, internexin and synaptotagmin I. Proteins implicated in the folding were also modulated, for instance HSP60 was increased whereas HSP70 was decreased in the ischemia-reperfusion condition. Interestingly, HSP60, HSP70, HSP90 as well as TOAD-64 were already identified in a rat middle cerebral artery occlusion model [29], hence showing that our method and proteomic method enable to identify markers of ischemia. The biological network of the characterized markers was established using CytoScape shareware [30]. Interestingly, several markers were interconnected (Figure 1B) and highlighted the potential signaling pathways that are deregulated during ischemia. Thus, at the intersection of several markers are signaling pathways related to GLUT4/Slc2a4 glucose transporter, MAPK3/ERK1 signaling and HSP90 which together may contribute to change of markers expression. However, our principal objective herein was to isolate and identify few markers that were modulated following Atorvastatin or Fenofibrate treatments and more precisely, that preserve of were restored to control levels in ischemia-reperfusion pre-treated animals. In this latter category, we found the protein disulfide isomerase A3/Erp60, the Janus kinase 2 as well as markers found in neurodegenerative diseases such as Parkinson’s disease (α-synuclein and DJ-1/Park 7; Figure 1B). These markers were further investigated by Western-blotting.

### Western blot

#### Alpha-synuclein

**Figure 2:**
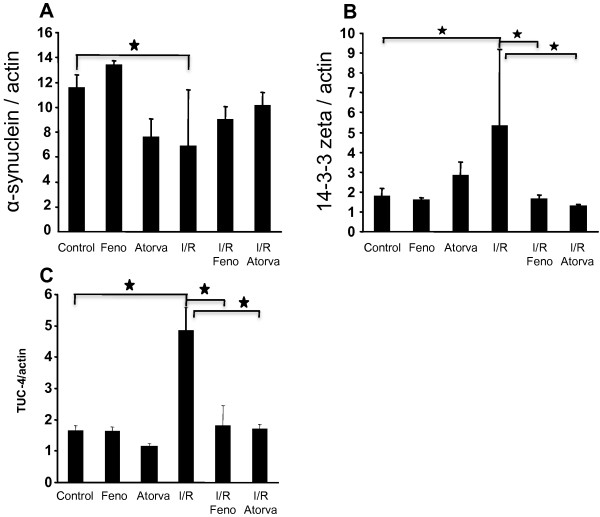
**Cortical expression of α-synuclein (2A), 14-3-3 Zeta (B) and TUC-4 (2C) in Control and treated rats.** Western blot and quantification of α-synuclein and 14-3-3 Zeta (KCIP-1) and TUC-4 expression in the brain cortex. Results are expressed as mean ± sem (n = 6 in each group). Statistical analysis using ANOVA followed by Fisher’s LSD test: ★ indicate a p value <0.05 vs. control group.

Referred to figure 2A, we observed a statistically significant decrease of the cortical expression of α-synuclein of atorvastatin-treated rats when compared to the control group. (OD ratio: Control group: 0.12 ± 0.01, Atorvastatin-treated group: 0.08 ± 0.02, p < 0.05). α-synuclein expression in I/R group was reduced (OD ratio: 0.07 ± 0.05) but not significantly when compared to control. With regards to fenofibrate-treated I/R rats and atorvastatin-treated I/R rats, we observed a similar tendency to a reduced expression of brain cortical α-synuclein expression. But the observed decrease in both fenofibrate-treated or atorvastatin group was not statistically significant (OD ratio respectively: 0.09 ± 0.01; 0.10 ± 0.01). α-synuclein can form intracellular aggregates when it is produce in excess leading to the formation of the Lewy bodies in the neuronal cytoplasm [31]. Accumulation of aggregated α-synuclein is one of the major causes of neuronal death in Parkinson disease and the loss of α-synuclein have been described in a model of perinatal hypoxia/ischemia [29] suggesting a sensitivity of dopaminergic neurons to hypoxia. Interestingly, atorvastatin showed its ability, for the first time, to lower α-synuclein expression. The mechanisms of the decrease are still elusive and several hypotheses are possible, including the interaction with other proteins or regulation of lipid metabolism. However, lowering α-synuclein could be protective toward the potential toxicity of α-synuclein in neurological disorders remains to be considered as a potential long-term treatment in Parkinson’s disease [32].

#### 14-3-3 Zeta

Concomitantly to the lowering of α-synuclein expression, we focused our attention on 14-3-3 ζ whose expression was modified in the cortex of rats constituting the experimental groups. As shown in figure 2B, 14-3-3 ζ expression was dramatically increased in the brain submitted to an ischemia-reperfusion procedure when compared to the control group (OD ratio: control group: 1.81 ± 0.37; I/R group: 5.34 ± 3.86; p < 0.05). To a lesser extent, we observed a moderate increased expression of 14-3-3 ζ when the rats were submitted to a pre-treatment by atorvastatin during 14 days before I/R (OD ratio: control group: 2.87 ± 0.67), which was not statistically significant. However, a 14 days fenofibrate treatment had no significant effect on 14-3-3 ζ expression level (OD ratio: 1.60 ± 0.09). Surprisingly, the effect of ischemia-reperfusion injury on 14-3-3 zeta increased expression was abolished under both fenofibrate or atorvastatin treatment. The hypolipidemic drugs treatment tends to diminish the expression of 14-3-3 ζ in the cortex (OD ratio: I/R Feno: 1.67 ± 0.18; 1.28 ± 0.07) when compared to control even if this lowering was not statistically significant. A potential role of 14-3-3 ζ in the formation of α-synuclein aggregates was suggested [33]. Even if speculative, we did not evaluate the co expression in the tissue of both α-synuclein and 14-3-3 ζ, our results tend to show an inverse correlation between the expression of α-synuclein and 14-3-3 ζ. The increase of 14-3-3 ζ after ischemia-reperfusion injury may constitute a mechanism of defense against the toxicity of oxidative stress induced protein modification, such as the oxidized α-synuclein [34]. The mechanism of induction of 14-3-3 ζ by atorvastatin therapy remains unknown. One of the limits of our work is the impossibility to identify the potential role of 14-3-3 ζ in the atorvastatin effect. However, a growing body of evidence suggest that 14-3-3 ζ could be a surrogate marker of brain injury; the level of 14-3-3 ζ is increased in cerebrospinal fluid of patient with Creutzfeld-Jacob disease or Seizure [35]. Additionally, an increased level 14-3-3 ζ in the cerebrospinal fluid from patients who had been operated of aortic aneurysm has been described and suggested to be central nervous markers of ischemic conditions [36]. The normal level of 14-3-3 ζ is restored in ischemia-reperfusion after pre-treatment with both atorvastatin and fenofibrate therefore suggesting that both drugs have potential neuroprotective effect and may more rapidly limit the neuronal injury induced after ischemia. A longitudinal study of patients treated with lipid lowering drugs and who faced an ischemic episode would be useful to acknowledge the potential neuroprotective effect of such drugs.

#### TUC-4

TUC-4 (dihydropyrimidinase-like 3, also referred to as Ulip1 or CRMP4 or TOAD) expression was increased in I/R condition. The protein TUC-4, as referred to Figure 2C showed a dramatic cortical increased expression when compared to the control group (OD ratio: Control group: 1.66 ± 0.16; I/R group: 4.84 ± 0.74; p < 0.05). In the condition used for this study, a 14 days pre-treatment by fenofibrate had no effect on the basal level of TUC-4 in the cortex of the animals (OD ratio: 1.63 ± 0.14) whereas atorvastatin showed a tendency to decrease TUC-4 expression in the cortex (OD ratio: 1.14 ± 0.10), even if this lowering did not reach the statistical significance threshold. TUC-4 belongs to a family of proteins expressed during neurogenesis, especially during the emerging cone setting and the axonal growth [37]. TUC-4 expression could rise in case of axonal damage [38]. In our work, we showed a dramatical increase of TUC-4 expression after ischemia-reperfusion injury. This result is consistent with the knowledge concerning TUC-4 and the neurogenesis after stroke [38]. However, the lack of effect of atorvastatin on TUC-4 expression was surprising, as atorvastatin is known to induce neurogenesis and synaptogenesis [39]. This result may be due to a difference in the treatment period, the treatment start point or the dose of atorvastatin. In our work, the dose corresponds to 15 mg/kg/day. In the work of Chen et al., the highest dose was 8 mg/kg/day and was the less effective. Interestingly, the administration of atorvastatin or fenofibrate before the ischemia-reperfusion injury leads to a normalization of the TUC-4 expression. The mechanisms of interaction between the drugs and ischemia-reperfusion injury are elusive and difficult to explain. The abolition of ischemia-reperfusion injury induced-mediators production by neuroprotective drugs is a hypothesis among others. Some investigations are needed to evaluate the relevance of such a result in stroke therapy.

#### PDI

**Figure 3:**
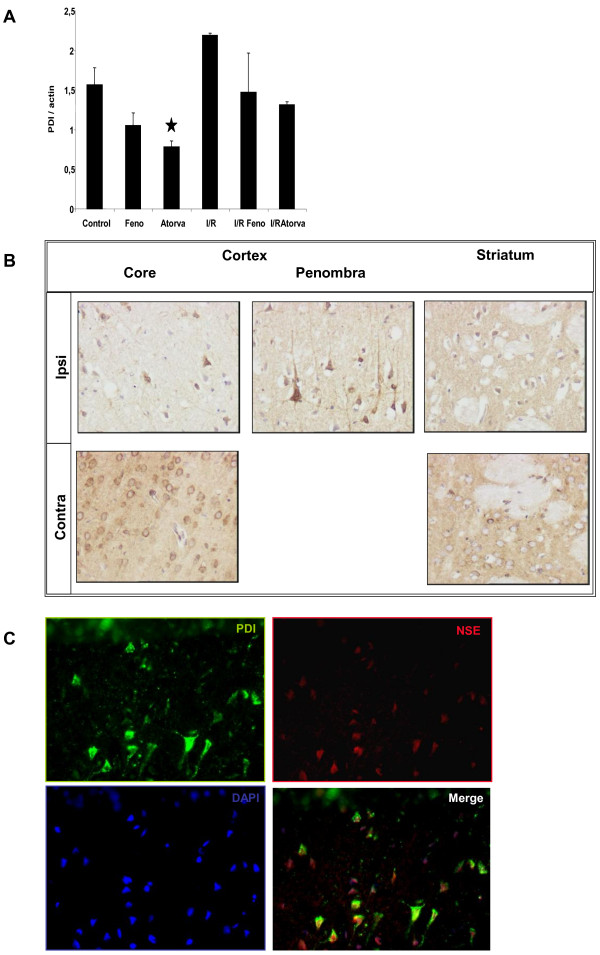
**Expression of Protein Disulfide Isomerase in the rat brain.** 3A Cortical expression of Protein Disulfide Isomerase (PDI, Erp 60) in the different groups. Results are expressed as mean ± sem (n = 6 in each group). Statistical analysis using ANOVA followed by Fisher’s LSD test: ★ indicates a p value < 0.05 vs Control group. 3B Expression of PDI in the cortex and striatum 24 hours after reperfusion. PDI is expressed under basal condition (contra) and only found in pycnotic cell of the penombra under ischemia-reperfusion injury. Striatum cells had a lower level of expression when compared to cortical cell. 3C The PDI expressing cell were identified as neuron using immunofluorescent double labelling with an NSE antibody.

Found during the proteome exploration, the expression variation of Protein Disulfide Isomerase A3 (58 kDa isoform, Erp60) (PDI) was studied by western blot (Figure 3A). The PDI is expressed in the cortex of the rat at a basal level. A 14 days fenofibrate treatment induced a slight but not statistically significant decrease expression of PDI (OD ratio: Control group: 1.57 ± 0.22; fenofibrate: 1.05 ± 0.16). A 14 days Atorvastatin treatment was more effective and induced a dramatic decrease of expression of PDI when compared to control (OD ratio: 0.78 ± 0.08, p < 0.05). Following Ischemia-Reperfusion injury, the PDI expression was enhanced in the cortex even if the statistical significance threshold was not reached (OD ratio: 2.18 ± 0.03). This elevation of PDI expression is reported to be lowered by anti-inflammatory treatment [40]. The hypolipidemic drug treatment before ischemia-reperfusion injury had a lowering effect on the ischemia-reperfusion injury induced PDI surged expression. Both fenofibrate and atorvastatin pre-treatments maintained a PDI expression comparable to that observed in control condition but statistically different from the ischemia-reperfusion group (OD ratio of fenofibrate and atorvastatin of 1.48 ± 0.5 and 1.32 ± 0.04, respectively; p < 0.05).

To further study the PDI expression following ischemia-reperfusion, we focused on cellular expression of PDI in the brain of rat as shown in figure 3B. Interestingly, PDI is expressed preferentially in the cortex of rat, when compared to other brain regions, especially the striatum. In the basal condition (Contra), PDI positive cells showed a strong staining of the cell soma close to the nucleus. Such a localization of the staining is compatible with an endoplasmic reticulum subcellular localization of the protein. After the ischemia-reperfusion injury, the PDI labelling was modified. In the core of the lesion, where necrotic death happens very early, PDI staining disappeared within the cells. In sharp contrast, in the penumbra, a robust PDI staining was essentially observed in pycnotic cells. The subcellular localization differed from that observed in control condition. The PDI was found in the soma and neuritic extensions. To identify the cellular type of PDI positive cells, we performed a co-immunostaining with the neuronal marker Neuron Specific Enolase (NSE), as illustrated in figure 3C. Interestingly, the PDI positive cells of the penumbra were also NSE positive, demonstrating that neurons are expressing the PDI. Together, Western-blot and immunohistochemistry analyses suggested that enhanced expression of PDI is resulting from reactive neurons in the penumbra whereas the core zone of ischemia, there is a loss of PDI expression. However, in the present study the effect of PPARα agonists was likely not associated with an anti-inflammatory effect [41] since inflammatory markers were not herein identify. However, our study suggests together with that of Llorente and collaborators that multiple therapeutic pathways may regulate the expression of PDI, and that the repression of PDI expression or activity may be show promising therapeutic in ischemia reperfusion. Thus, recent studies have suggested a protective effect PDI ligands such as 4-hydrobenzyl alcohol against brain ischemia reperfusion [41], [42] supporting the idea that PDI is likely an interesting pharmacological target for neuroprotection, an further study are certainly necessary to detail the underlying molecular mechanism.

## Conclusions

The present study, using a combined pharmacological and proteomic approach, aimed to decipher with the major signalling pathway and molecular target useful for neuroprotective purposes in brain ischemia/reperfusion. Using two drugs shown to have potential neuroprotective effects, we thus showed that signalling pathways related to unfolded protein response could be represent interesting pharmacological targets such as PDIA3/Erp60.

## Materials and methods

### Animals and treatments

Male Wistar rats were obtained from Charles River Laboratories (Charles River Laboratories, Le Bois d’Arbresle, France). The rats weight range from 280 to 300 g at the beginning of the experiments. The rats had a free access to food and water during the treatment period. All animal experiments were performed within the frameworks of the French legislation and received the approval of the local ethic committee (Ethic committee of Nord-Pas-de-Calais for animal experimentation, CEEA, Lille, France). A group of Wistar rats (n = 12) was submitted to a treatment by PPARα agonist. This group was fed with a 0.2% fenofibrate-enriched diet during 14 days. A second group (n = 12) was submitted to a treatment by statin. Animals (n = 12) were fed during 14 days with 0.1% atorvastatin-enriched diet. At the same time, a control group was fed with standard diet. At the end of the two weeks period, one half of the rats of each group was submitted to a middle cerebral artery occlusion. For proteomics analyses, 6 animals in each group were used. Animals were sacrificed by injection of a lethal dose of pentobarbital, followed by a transcardial perfusion of ice cold 10 mM phosphate buffered saline (PBS, pH 7.4). Brain were harvested, immersed in ice cold PBS, forebrain and cerebrum were discarded, cortex and striatum were dissected, sliced in small pieces and frozen into liquid nitrogen. Brain samples were stored at -80°C until use. For immunohistochemistry analyses, 6 animals in each group were used. The animals were transcardialy perfused with cold heparinized (500 U/L) saline solution. Thereafter, animals were perfused with 4% paraformaldehyde (PFA) in PBS (pH 7.4). Brains were removed and post-fixed in the same fixative solution during 4 hrs at 4°C, then processed for paraffin embedding and cut into 6 μm thick sections.

### Middle cerebral artery occlusion method

Animals were anesthetized with chloral hydrate (300 mg/kg, I.P.) (Sigma-Aldrich, Saint Quentin Fallavier, France). Cerebral infarcts were produced by a 60 min medial cerebral artery (MCA) occlusion followed by a 24 h-reperfusion period [43]. We introduced into the internal carotid artery a 4–0 nylon monofilament suture with its tip rounded by flame heating. Under surgical microscope control, the suture was gently advanced into the internal carotid artery to occlude the origin of MCA (25 to 30 mm distal to the carotid bifurcation). After 60 min, the suture was carefully removed to allow reperfusion. A rectal probe was inserted and the core temperature was maintained at ~ 37°C by the use of a heating pad and a heating lamp.

### Two-dimensional gel electrophoresis

For 2D analysis, frozen brain samples were homogenized in 2D sample buffer using Wheaton glass homogenizer in 2D sample buffer containing 7 M urea, 2 M thiourea, Triton X-100 4% v/v, DTT 20 mM. 400 μg of protein were diluted to a final volume of 400 μL and 0.6% carrier ampholytes (pH 4–7) and 10 mM Tris (final dilution) were added immediately prior to protein load. IPG isoelectrofocusing (IEF) was carried out in a Protean IEF cell (Biorad, Hercules, CA), using a 18 cm pH 4–7 Amersham ready strip. The IPG strips were rehydrated overnight with the protein lysate. For subsequent IEF, voltage was increased gradually to 10.000 V to reach a total of 60.000 V/hr. Immediately after focusing, strips were equilibrated for 3×15 minutes in a solution containing 50 mM Tris, 2% SDS, 20% glycerol, and 1% DTT w/v, and 0.01% w/v Bromophenol blue as tracking dye. SDS-PAGE was performed in a Protean IIXi Cell (Biorad) on a 12% polyacrylamide gel, until dye track reach the end of the gels. Gels were Coomassie’s Brillant Blue-stained for a first analysis, followed by a silver-staining procedure. Thereafter, stained gels were digitized and processed using Melanie III two-dimensional gel analysis software (Genebio, Geneva, Switzerland) for spot detection and analysis. Spots were edited manually to remove technical artefacts such as gel distortion and polypeptides were identified by mass spectrometry as previously described [44].

### Western blot

For western blot analysis, frozen brain samples were sonicated in RIPA lysis Buffer (150 mM NaCl, 6 mM sodium deoxycholate, 1 mM EGTA, 1 mM Igepal CA-630, 1% ICN proteases inhibitors cocktail kit, Tris–HCl 50 mM pH 7.4), mixed with Laemmli reducing sample buffer and heated at 100°C for 5 minutes. 50 μg of protein were loaded onto a 4-12% SDS-PAGE gradient gel and submitted to an overnight electrophoresis at 20 mA per gel. At the end of the procedure, proteins were transferred on a nitrocellulose membrane. After blocking the non specific interaction sites using 5% milk in 20 mM Tris buffered saline 0.05% Tween-20 (TBST), pH 7.4, membrane was incubated overnight at 4°C with specific primary antibodies solution at 1 μg/mL TBST 5% milk (mouse anti-PDI (clone 1D3), SPA-891 from Stressgen, mouse anti-alpha-synuclein BD610786 from BD bioscience, rabbit anti- 14-3-3 Zeta SC-1019 from SantaCruz Biotechnologies, rabbit anti-TUC-4 AB5454 and pan-TUC ABN108from Chemicon and mouse anti-actin A2413 from Sigma). Thereafter, antibodies were detected with species-specific HRP coupled secondary antibodies (Goat anti-rabbit IgG-HRP AP132P, Goat anti-mouse IgG-HRP AP127P, from Chemicon) 1/50000 in TBST. Detection was performed using ECL chemiluminescent system (Amersham) and LAS3000 lumi-imager (FujiFilm, Japan).

### Immunohistochemistry

Paraffin embedded sections were successively deparaffinized with toluene and rehydrated using a classical procedure of successive ethanol decreasing grade baths. Sections were permeabilized by immersion in 0.3% Triton X-100 in PBS (pH 7.4). Thereafter, endogenous peroxidases were blocked with 0.3% H_2_O_2_ and 10% methanol in PBS (pH 7.4). Protein nonspecific binding was blocked with 5% normal horse serum in PBS (pH 7.4). Sections were then incubated over night at 4°C with a mouse anti-Protein Disulfide Isomerase monoclonal antibody (5 μg/mL final dilution, mouse anti-PDI, SPA-891 from Stressgen) and/or a rabbit anti-Neuron Specific Enolase (5 μg/mL, NA 1247 from Biomol International) in PBS. Antibodies were visualized with an anti-goat Vectastain ABC Elite kit (Vector laboratories) and DAB/H_2_O_2_ exposure (Sigma Fast DAB tablets set, Sigma) according to the manufacturers’ instructions. For double labelling, antibodies were visualized using an Alexa Fluor-422 labelled goat anti-mouse antibody (A11001) and an Alexa Fluor 565 labelled goat anti-rabbit antibody (A11011) (1/500 in PBS, Molecular Probes).

### Statistical analysis

For statistical data comparison, a standard software package (PRISM GraphPad for Windows) was used. Variables were compared between different groups with a one-way variance analysis followed by least significance difference tests. Statistical tests are considered as significant when p ≤ 0.05. All values are given as mean ± sem.

### Bioinformatics analysis

The biomolecular interactome and gene ontology analysis was performed using Cytoskape shareware [30], [45].

## Abbreviations

2D: Two-dimensional; AKT: Protein kinase B; DTT: Dithithreitol; EGTA: Ethylene glycol tetraacetic acid; ERK: Extracellular signal-regulated kinase; HMG-CoA: Hydroxyl methyl coenzyme A; HSP: Heat-shock protein; I/R: Ischemia/reperfusion; OD: Optical density; PBS: Phosphate buffer saline; PDI: Protein disulphide isomerase; PFA: Paraformaldehyde; PI3K: Phosphoinositide 3-kinase; PKC: Protein kinase C; PPAR: Peroxisome proliferator-activated receptor; RIPA: Radio immunoprecipitation assay; TBS: Tris-buffer saline; TOAD-64: Turned on after division/Ulip/CRMP.

## Competing interests

Authors declare no competing interests.

## Authors’ contributions

PG, VV, HD, AG have perform the experiments, PM, LB, NS and RB have contributed to the designed of the study and contributed intellectually, PG and NS have analysed the data and PG and NS have written the manuscript. All authors read and approved the final manuscript.
